# Hydrogel Swelling-Mediated Strain Induces Cell Alignment at Dentin Interfaces

**DOI:** 10.1021/acsbiomaterials.2c00566

**Published:** 2022-07-06

**Authors:** David Fraser, Tram Nguyen, Alexander Kotelsky, Whasil Lee, Mark Buckley, Danielle S. W. Benoit

**Affiliations:** † Eastman Institute for Oral Health, Department of Periodontology, University of Rochester Medical Center, Rochester, New York 14620, United States; ‡ Translational Biomedical Science, University of Rochester Medical Center, Rochester, New York 14642, United States; § Department of Biomedical Engineering, University of Rochester, Rochester, New York 14627, United States; ∥ Department of Pharmacology & Physiology, University of Rochester Medical Center, Rochester, New York 14642, United States; ⊥ Center for Musculoskeletal Research, University of Rochester Medical Center, Rochester, New York 14642, United States; # Department of Chemical Engineering, University of Rochester, Rochester, New York 14627, United States; ∇ Materials Science Program, University of Rochester, Rochester, New York 14627, United States

**Keywords:** cell alignment, periodontal ligament cells, dentin, hydrogels, swelling, strain

## Abstract

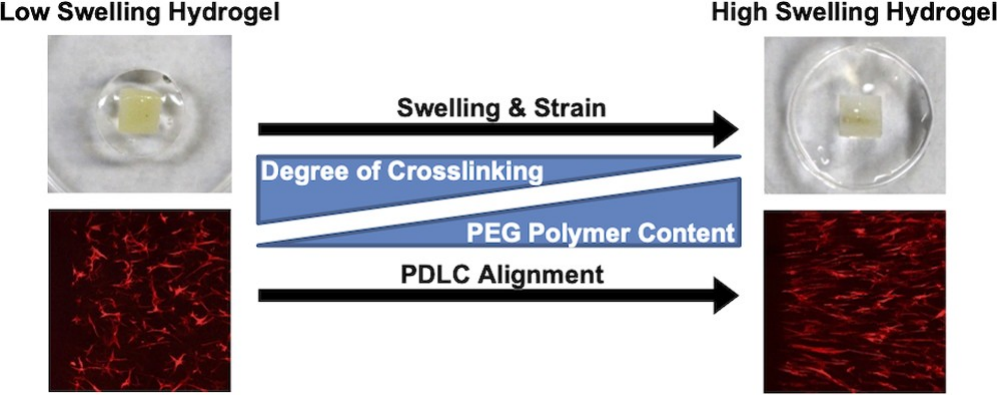

Cell and tissue alignment is a defining feature of periodontal tissues. Therefore, the development of scaffolds that can guide alignment of periodontal ligament cells (PDLCs) relative to tooth root (dentin) surfaces is highly relevant for periodontal tissue engineering. To control PDLC alignment adjacent to the dentin surface, poly(ethylene glycol) (PEG)-based hydrogels were explored as a highly tunable matrix for encapsulating cells and directing their activity. Specifically, a composite system consisting of dentin blocks, PEG hydrogels, and PDLCs was created to control PDLC alignment through hydrogel swelling. PDLCs in composites with minimal hydrogel swelling showed random alignment adjacent to dentin blocks. In direct contrast, the presence of hydrogel swelling resulted in PDLC alignment perpendicular to the dentin surface, with the degree and extension of alignment increasing as a function of swelling. Replicating this phenomenon with different molds, block materials, and cells, together with predictive modeling, indicated that PDLC alignment was primarily a biomechanical response to swelling-mediated strain. Altogether, this study describes a novel method for inducing cell alignment adjacent to stiff surfaces through applied strain and provides a model for the study and engineering of periodontal and other aligned tissues.

## Introduction

Alignment of collagen fibers is a defining feature of fibrous tissues, including ligaments and tendons, that form insertion sites with mineralized tissues. The periodontal ligament (PDL) is one such tissue, connecting the tooth root cementum and alveolar bone through aligned collagen (Sharpey’s) fibers, which allows the transfer of functional forces from teeth to the surrounding tissues.^1^ This periodontal complex is destroyed by periodontitis, and rebuilding the PDL–cementum and PDL–alveolar bone entheses continues to be a clinical challenge. A wide variety of biomaterials and scaffolds have been designed to direct the orientation of periodontal ligament cells (PDLCs), based on the premise that cell alignment can precede the formation of aligned collagen fibers.^2,3^ These approaches typically employ organized fibers, struts, or channels to induce cell alignment through contact guidance.^4^ Cell alignment can also be achieved on nonpatterned substrates or within amorphous materials through the application of external mechanical forces.^5^ Despite these advances, significant challenges remain in coordinating PDLC alignment relative to dentin surfaces as well as understanding the mechanisms driving aligned PDL formation and repair, a process likely driven by biomechanical strain at the cell and tissue level.^6^

Hydrogels are highly hydrated networks formed from natural (e.g., collagen or fibrin) or synthetic polymers. Multiarm poly(ethylene glycol) (PEG)-based polymers are a versatile material for creating hydrogels with defined biological and mechanical properties.^7^ Tethering of functional groups, such as the cell-adhesive peptide RGD, to PEG arms and the use of matrix-metalloproteinase (MMP)-degradable peptide cross-linkers allows cells to bind and spread within the hydrogel matrix. This modular system also affords control over mechanical properties such as stiffness through varying PEG polymer content and the degree of cross-linking (ratio of PEG arms to cross-linker arms). These two parameters are also intrinsically linked with hydrogel swelling. Once formed and placed in an aqueous solution, hydrogels swell after formation until an equilibrium is reached between the elastic forces of the cross-linked polymer chains and the mixing forces of the solvent and hydrophilic polymer chains.^8^ For hydrogels formed with multiarm PEG macromers, increasing the polymer content while maintaining or decreasing the degree of cross-linking increases the hydrogel swelling via the introduction of additional free PEG arms, while increasing the degree of cross-linking counteracts solvent-mixing forces and reduces swelling. Hydrogel swelling as a tunable property is well described for biomedical applications such as drug delivery,^9^ but few studies have investigated the impact of swelling on cell morphology or function within hydrogels.^10,11^ Thus, the purpose of this study was to determine if hydrogel swelling could be harnessed to alter PDLC alignment, using PEG hydrogels and dentin substrates to model the PDL and tooth root.

## Materials and Methods

### Material Synthesis

Eight-arm PEG hydroxyl (JenKem Technology, TX) was functionalized with norbornene (5-norbornene-2-carboxylic acid, Alfa Aesar, MO) via *N*,*N*′-dicyclohexylcarbodiimide (DCC) coupling using a previously described method.^12^ Functionalization was determined via ^1^H NMR [CDCl_3_]: δ = ∼3.6 for PEG ether protons, δ = 5.9–6.3 for norbornene vinyl protons, with PEG macromers having ≥90% functionality used for all experiments. The cell-adhesive peptide CGRGDS (RGD) was synthesized using a Liberty 1 microwave-assisted peptide synthesizer (CEM, NC) as previously described,^13^ and the MMP-degradable peptide cross-linker GKKCGPQGIWGQCKKG was purchased from Genscript (NJ). Peptides were dissolved in phosphate-buffered saline (PBS) and stored at −80 °C. Free thiol concentrations of each peptide batch were measured using Ellman’s reagent (Fisher Scientific). The photoinitiator lithium phenyl-2,4,6-trimethylbenzoylphosphinate (LAP) was synthesized using established methods.^14^

### Hydrogel Formation and Characterization

Hydrogels were formed by suspending PEG-norbornene, and RGD and cross-linker peptides in a range of concentrations (Table 1) in Dulbecco’s phosphate-buffered saline (PBS, Gibco) together with 0.05 wt % LAP. Thirty microliters of hydrogel solution was pipetted into 1 mL cylindrical syringe molds with a 4.3 mm diameter and exposed to UV light (5 mW/cm^2^, 365 nm) for 3 min. After formation, hydrogel dimensions were measured with digital calipers (General Tools) to determine initial volumes. Hydrogels were then placed in PBS for 24 h to reach equilibrium swelling, after which the dimensions were again measured to determine the swollen volume. Swollen hydrogel mass was then recorded, after which hydrogels were frozen and lyophilized to determine the dry hydrogel mass. Elastic modulus was measured on a separate set of hydrogels using an MTS test frame (MN) equipped with a 5 N load cell, compressing between 5 and 10% of initial hydrogel height at a rate of 0.1 mm/s.

**Table 1:** Hydrogel Compositions

degree of swelling	PEG content (wt %)	degree of cross-linking (%)	cross-linked arms, initial (mM)	cross-linked arms, swollen^a^ (mM)	free arms, initial (mM)	free arms, swollen^a^ (mM)	RGD concentration, initial (mM)	RGD concentration, swollen^a^ (mM)	PDLC concentration, initial (cells/mL)	PDLC concentration, swollen^a^ (cells/mL)
low (L)	2.5	85	7.4	7.7 ± 0.5	2.6	2.7 ± 0.2	1	1.0 ± 0.07	2 × 10^6^	2.1 ± 0.1 × 10^3^
moderate (M)	4	65	9.0	6.1 ± 0.2	7.0	4.7 ± 0.1	1.5	1.0 ± 0.03	3 × 10^6^	2.0 ± 0.1 × 10^3^
high (H)	7	45	11.0	5.3 ± 0.2	17.0	8.3 ± 0.3	2	1.0 ± 0.04	4 × 10^6^	1.9 ± 0.8 × 10^3^

a Estimated using fold-change increase in hydrogel volume from initial to swollen state.

Fold-change hydrogel volume was calculated as the ratio of the swollen hydrogel volume (*V*_s_) to the initial hydrogel volume (*V*_i_)
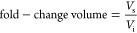
1Mass swelling ratio (*q*) was determined from the swollen hydrogel mass (*M*_s_) and dry hydrogel mass (*M*_s_)^15^
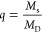
2Volumetric swelling ratio (*Q*_V_) was calculated as the inverse of the swollen polymer fraction *v*_2,S_, where *V*_D_ is the volume of the dry polymer^16^
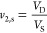
3
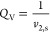
4Hydrogel mesh sizes were estimated from *q* using a modified Flory–Rehner equation as described previously.^17^

### Isolation of PDLCs and Preparation of Block Materials

Human periodontal ligament cells (PDLCs) and dentin blocks were obtained from extracted 3rd molars following informed consent (URMC Research Subjects Review Board protocol 00072932). PDL tissues were removed from the middle third of roots, minced into 0.5 mm pieces, digested for 1 h in PBS containing collagenase type 1 (900 U/mL, Gibco) and dispase type II (2.3 U/mL, Sigma), and then passed through 70 μm cell strainers to obtain PDLCs. Single-cell solutions were plated in six-well plates at 1000 cells/cm^2^ in αMEM (Gibco) supplemented with 20% fetal bovine serum (FBS), and 1× antibiotic–antimycotic (Gibco). After 7–10 days, adherent PDLCs were detached with 0.05% trypsin/ethylenediaminetetraacetic acid (EDTA) (Gibco) for expansion or cryopreservation. At passage 3, PDLCs were characterized for cell surface marker expression via flow cytometry to confirm mesenchymal origin. PDLCs from a single donor at passages 3–6 were used for all experiments. After PDL harvest, all remaining soft tissues and cementum were removed from tooth roots using curettes and a Dremel to produce a uniform dentin surface. A trephine (Fine Science Tools) was used to obtain dentin cylinders (1 mm radius and 2 mm height) and a diamond-coated disk (Brasseler) was used to create dentin cubes (2 mm length, 2 mm width, 2 mm height). Blocks were also created from human bone (Anatomy Gifts Registry) and polystyrene tissue culture plates. Blocks were stored in 70% ethanol and then sterilized via autoclave prior to use.

### Formation and Monitoring of Dentin–Hydrogel Composites

A dentin–hydrogel composite system was created by placing dentin blocks in the center of hydrogel molds prior to the addition of hydrogel solution. PDLCs were suspended in hydrogel solutions at low concentrations (2–4 × 10^6^ cells/mL) to ensure minimal cell-cell contact at both formation and after equilibrium swelling (Table 1). Composites were then removed from molds and cultured in media (MEMα with 10% and 1× antibiotic–antimycotic, Gibco) for up to one week. PDLC viability within composites was monitored using a Live/Dead staining kit (Invitrogen, MA). PDLC proliferation was analyzed using PrestoBlue (Invitrogen). Immediately after formation, at equilibrium (24 h after formation), and at one week, composites were placed in a fresh solution of 10% PrestoBlue in media and cultured for 1 h, after which a portion of the media was transferred to 96-well plates to read fluorescence at 560/590 nm. Relative fluorescence units for each composite was normalized to values at formation to calculate proliferation.

### Analysis of PDLC Alignment and Aspect Ratio

After 1 week of culture, composites with dentin cubes were rinsed twice in PBS, fixed in 4% paraformaldehyde for 15 min, rinsed twice in PBS, and stained with Alexa Fluor 568-tagged phalloidin (Invitrogen) overnight. Images were acquired on a spinning disk confocal microscope (Andor, Oxford Instruments) using 10× magnification at a depth of 200–300 μm from the hydrogel surface. Fusion software stitching (Andor, Oxford Instruments) was used to create whole composite images. PDLC alignment was quantified using the Directionality function in ImageJ. Four sections (1000 μm × 2000 μm) of each whole composite image were selected corresponding to each side of the dentin block. Each section image was then rotated so the edge of the dentin block was at the top of the image, converted to 8 bit, and then divided into 1000 μm^2^ wide near (close to dentin) and far sections. The Directionality tool was then used separately for each section with the Fourier component method and nbins:45. The mean value for each bin in the near or far region for the combined four sides was used as the value for each whole composite bin. These measurements were repeated for three composites per condition to give the mean ± standard deviation direction for each bin. PDLC alignment was also quantified in four 500 μm^2^ wide consecutive sections moving away from the dentin block. The particle analysis tool in ImageJ was used to determine the mean PDLC aspect ratio, defined as the length of the major PDLC axis divided by the minor axis, in each 500 μm^2^ section.

### Analysis of PDLC Gene and Protein Expression

Composites with dentin cylinders were utilized to allow uniform isolation of the central region of aligned PDLCs in high-swelling composites with a 4 mm tissue punch. After one week of culture, hydrogels from complete low-swelling or sectioned high-swelling composites were digested with 1000 U/mL collagenase II in PBS. Isolated PDLCs were rinsed twice in PBS, then subjected to RNA isolation with TRIzol (Invitrogen), followed by transfer to spin columns (E.Z.N.A., Omega Bio-Tek) for rinsing and DNAse treatment. cDNA synthesis was performed using iScript (BioRad). qPCR was performed with primers for *Periostin* (*POSTN*; forward: GGAGGCAAACAGCTCAGAGT, reverse: AATCGCACCGTTTCTCCCTT), *collagen type 1 α 1* (*COL1A1*; forward: GCCAAGACGAAGACATCCCA, reverse: GGCAGTTCTTGGTCTCGTCA), and housekeeping gene *RPL32* (forward: CAACATTGGTTATGGAAGCAACA, reverse: TGACGTTGTGGACCAGGAACT), together with PowerUp SYBR green master mix (Applied Biosystems) at an annealing temperature of 60 °C. Relative gene expression was calculated using the Pfaffl method.^18^

For fluorescent staining of proteins, high-swelling hydrogel composites with dentin cubes were rinsed in PBS, fixed with 4% paraformaldehyde, and blocked with 5% bovine serum albumin (BSA) and 0.1% Triton X-100 for 1 h. Staining with primary antibodies (mouse anti-collagen type 1: ab6308, Abcam; rabbit anti-Periostin: ab14041, Abcam) was performed overnight at 4 C. After rinsing, fluorescent secondary antibodies (goat antimouse Alexa Fluor 488, Invitrogen; goat antirabbit Alexa Fluor 647, Invitrogen) were applied overnight at 4 C. Hydrogels were incubated with 500 nM 4′,6-diamidino-2-phenylindole (DAPI) for 1 h, rinsed three times, and then imaged at 40× magnification with z-stack images taken at 0.29 μm intervals. Maximum intensity projection images were created in ImageJ to visualize protein.

### Finite-Element Analysis (FEA)

Finite-element analysis (FEA) was performed in FEBio^19^ to simulate swelling of hydrogels with and without cylindrical dentin blocks and to compare the resulting radial tensile strains (i.e., radially measured first principal strains). Hydrogel swelling was induced by a 10 kPa effective fluid pressure prescribed on the hydrogel boundary. The hydrogel was modeled as a neo-Hookean biphasic material with Young’s modulus of 3.5 kPa and Poisson ratio of 0.49.^15^ A cylindrical dentin block (height: 2 mm, radius: 1 mm), embedded in the center of the hydrogel, was modeled as a neo-Hookean hyperelastic solid with Young’s modulus of 20 GPa and Poisson ratio of 0.3.^20,21^ Tensile strains observed in hydrogels with or without dentin cylinders were compared as a function of radial position from the center of the hydrogel or dentin cylinder. Note that the effective fluid pressure implemented in FEA was not experimentally measured; however, this quantity was consistently implemented in the two finite-element models, allowing fair comparisons of the tensile strains. In addition, the FEA did not allow simulation of hydrogel swelling beyond a 2 mm radius without distorting finite elements, leading to a lack of convergence. Nevertheless, it was anticipated that the tensile strain gradient would be present at greater fold-changes in swelling volume and would also plateau to a level seen in hydrogels alone at an increased radius.

### Statistical Analysis

All data were analyzed and visualized using Prism (GraphPad, CA) and presented as mean ± standard deviation. Unpaired *t*-tests were used to compare two independent groups and analysis of variance (ANOVA) with Tukey posthoc corrections for comparing three or more independent groups. A *p* value less than 0.05 was considered statistically significant.

## Results

**Figure 1:**
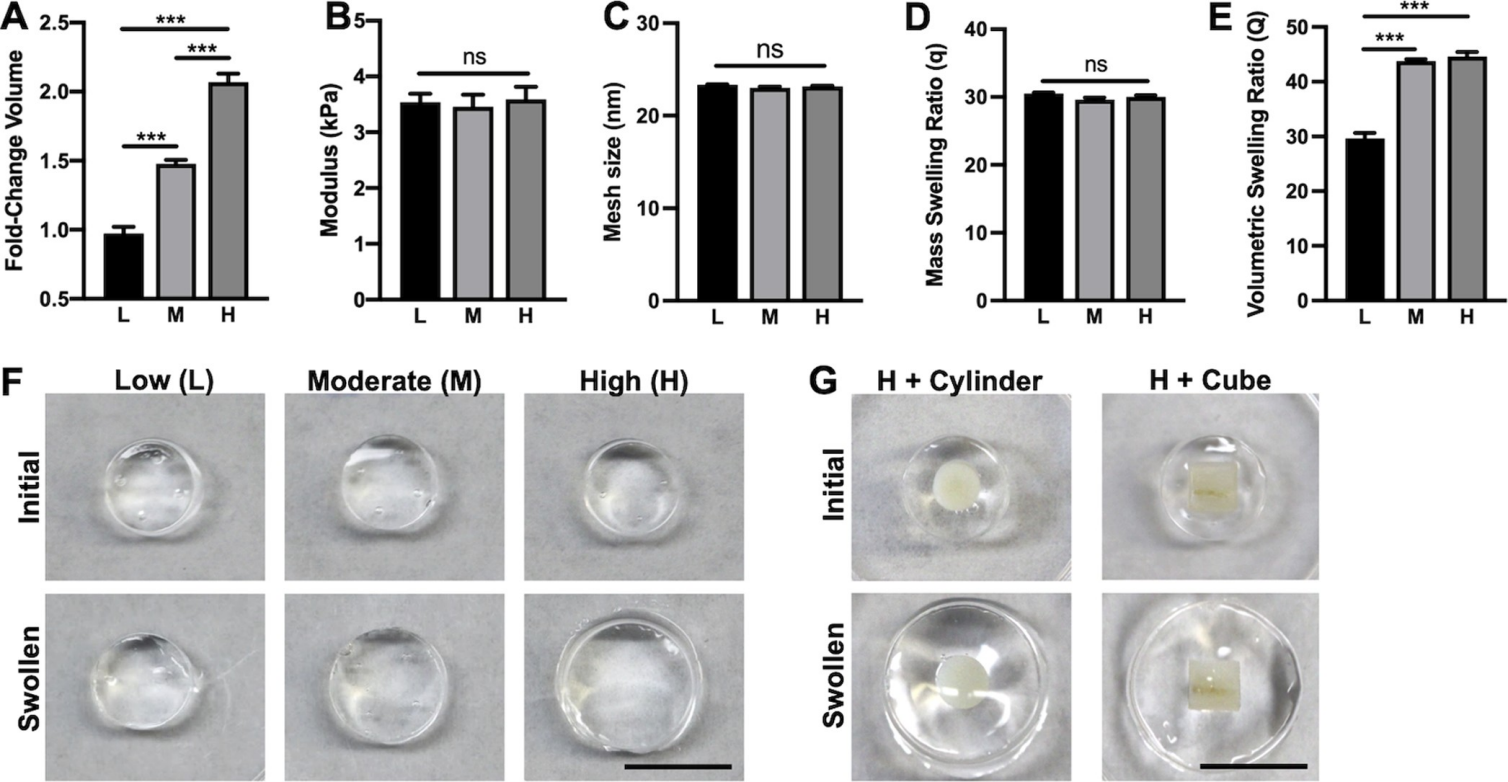
Swelling and mechanical properties of low (L), moderate (M), and high swelling (H) hydrogels. (A) Fold-change in hydrogel volume from after formation (initial) to equilibrium swelling (swollen). (B) Elastic modulus of equilibrium swollen hydrogels. (C) Hydrogel mesh size. (D) Mass swelling ratio. (E) Volumetric swelling ratio. *N* = 3 hydrogels. Results are given as mean ± standard deviation. One-way ANOVA with Tukey posthoc, ***p < 0.01, ns: not significant. (F) Representative photographs of hydrogels in each swelling group after formation (initial) and after reaching equilibrium swelling (swollen). (G) Initial and swollen high-swelling hydrogel composites with dentin cylinder or cube. Scale bars are 5 mm.

Three hydrogel conditions were identified with low, moderate, and high-swelling behavior (Figure 1A). Notably, all hydrogels had similar elastic modulus and mesh size at equilibrium (Figure 1B,C), suggesting that swelling would be the primary factor influencing cellular behavior from that point. Calculations of the hydrogel swelling ratio, based on either swollen hydrogel mass^15^ or volume^22^ and dry polymer mass, did not reflect the change in volume between initially formed and swollen hydrogels (Figure 1D,E), a finding that is likely due to the dry polymer mass of each type of hydrogel containing different proportions of cross-linked versus free PEG arms. Fold-changes in hydrogel volume between formation and equilibrium were used to set initial RGD and PDLC concentrations so that both factors would be similar after the initial swelling period (Table 1).

PDLCs were first suspended in PEG hydrogels without dentin blocks and cultured for 1 week at 37 °C. During this period, PDLCs spread throughout hydrogels with a randomly aligned, spindle morphology, with the degree of hydrogel swelling and any associated differences in the concentrations of cross-linked or free PEG polymer arms (Table 1) showing no impact on either PDLC spreading (aspect ratio) or relative alignment (Figure S1). Similar to hydrogels without dentin, hydrogel composites swelled within the first 24 h after formation, with PDLCs maintaining high viability (≥95%) and showing similar levels of proliferation through 1 week regardless of swelling degree (Figure S2).

**Figure 2:**
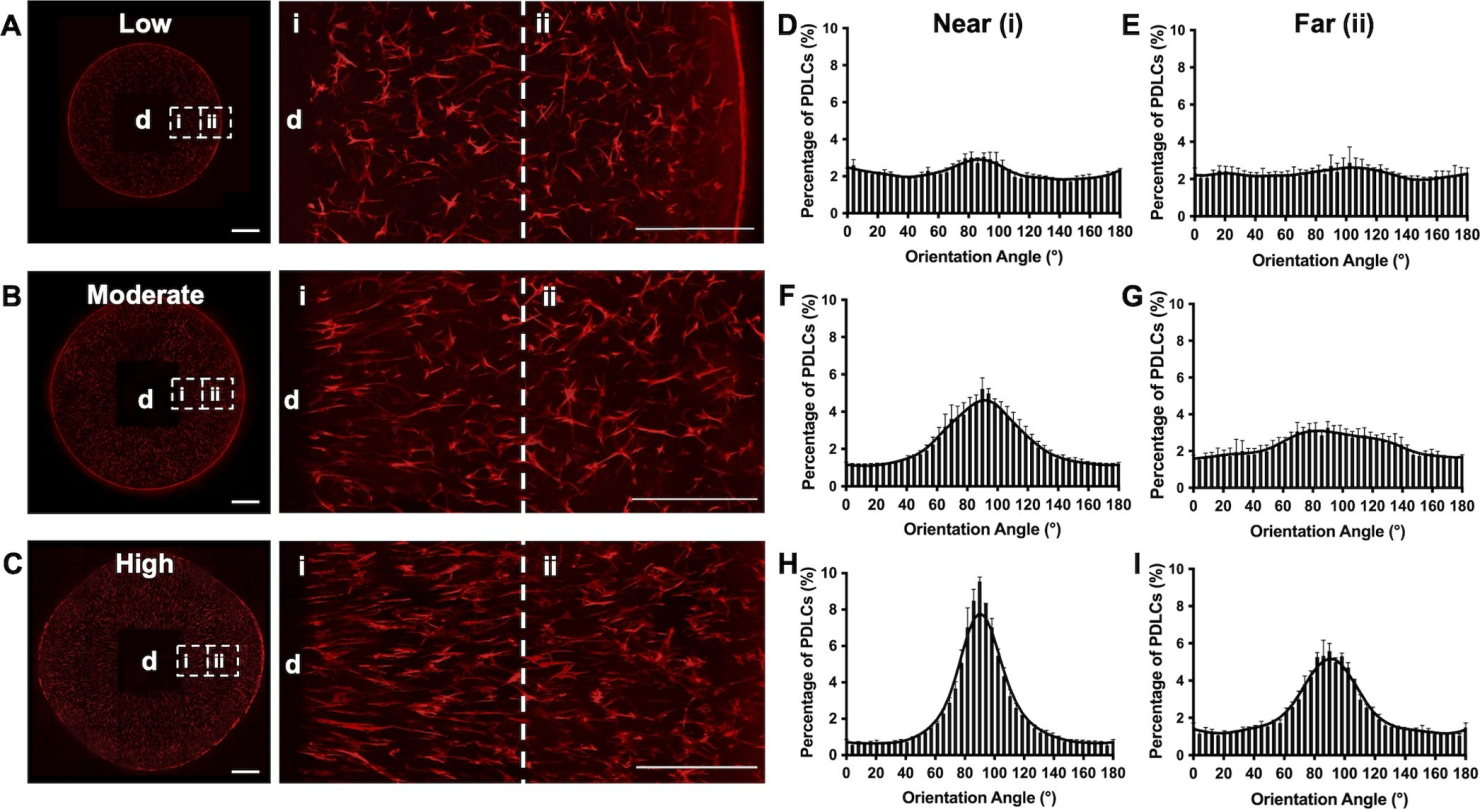
Low (A), moderate (B), and high (C) swelling hydrogel composites with dentin cubes (d) stained with Alex Fluor 568 phalloidin (red). Insets (i) and (ii) are representative images showing the near (i) and far (ii) regions where PDLC orientation was quantified. Histograms showing the percentages of PDLCs with indicated orientation angles relative to the dentin surface in near (D, F, H) and far (E, G, I) regions of low (D, E), moderate (F, G), and high swelling (H, I) composites. Bold lines are curves from locally weighted scatterplot smoothing (LOWESS) regression. *N* = 3 composites per group. Scale bars in the whole composite images are 1 mm. Scale bars in insets are 500 μm.

PDLCs in composites with low-swelling showed random alignment in all hydrogel regions (Figure 2A). In contrast, a striking PDLC alignment occurred adjacent to dentin surfaces as a function of the extent of hydrogel swelling, with the long axes of PDLCs adjacent to the blocks arranged perpendicular to the dentin surface (Figure 2B,C). Furthermore, both the degree of alignment, shown as a peak in percentage of PDLCs with relative orientation angles from 80 to 100°, and the extension of aligned PDLCs away from the dentin surface increased as swelling increased (Figure 2D–I). A similar relationship between swelling and PDLC alignment occurred at the surface of dentin cylinders (Figure S3) as well as in hydrogel–dentin composites created using half-cylinder and rectangular molds (Figure S4A,B). Bone blocks, polystyrene blocks, and human bone marrow stromal cells (BMSCs) (Lonza) were also substituted for dentin blocks and/or PDLCs. Cells aligned adjacent to each substrate within swollen hydrogels (Figure S4C–F), indicating that cell alignment may be a function of hydrogel swelling adjacent to a stiff object rather than specific characteristics of the PDLCs or dentin blocks.

**Figure 3:**
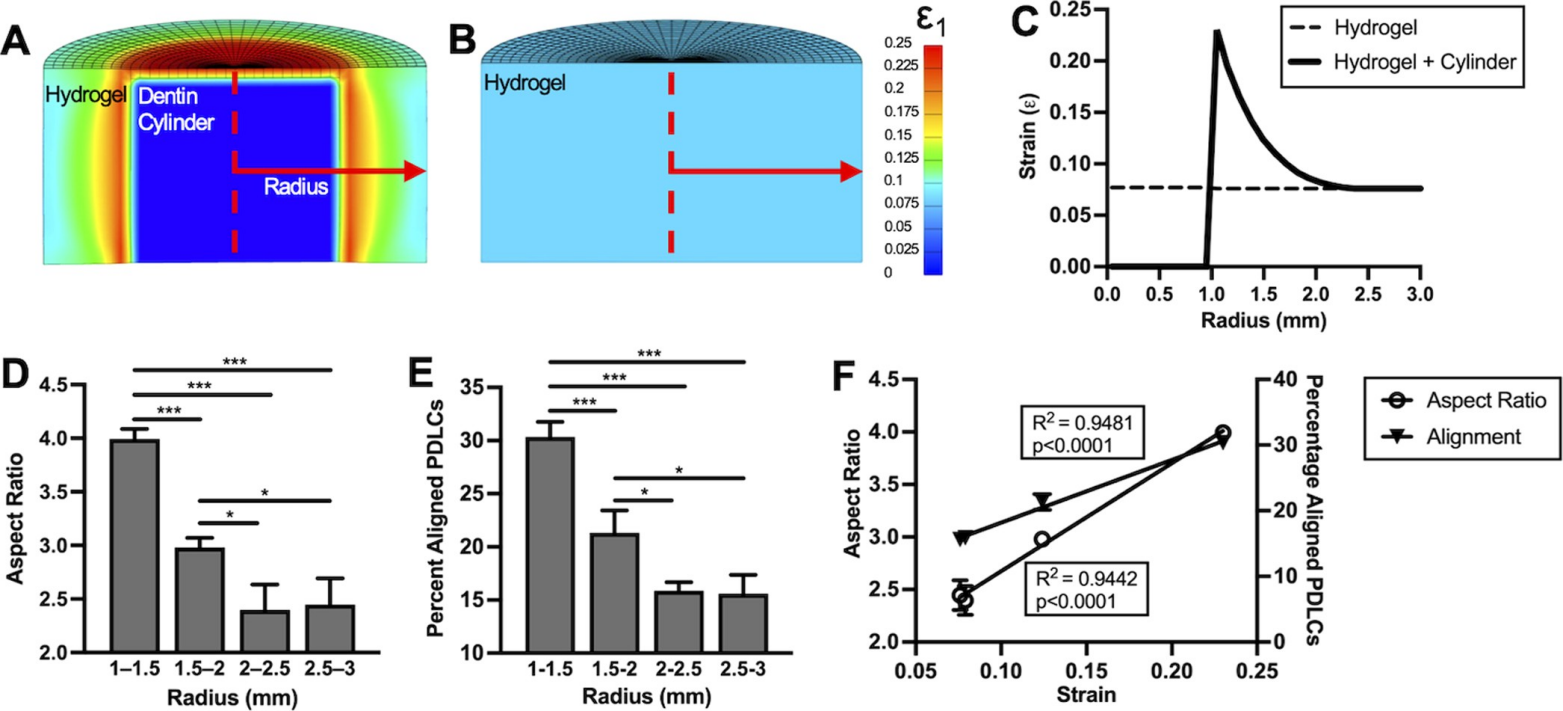
First principal strain (ε_1_) maps from the finite-element analysis of swollen hydrogel–dentin composites with a cylindrical block (A) and hydrogels alone (B). (C) Tensile strains as a function of radial distance from the center of a hydrogel–dentin composite or a hydrogel. (D) Mean aspect ratio of PDLCs in composites within the given radius. (E) Percentage of aligned PDLCs, defined as having an orientation angle within 80–100° of the dentin surface, within the given radius. *N* = 3 composites. One-way ANOVA with Tukey posthoc, *p < 0.05, ***p < 0.01. (F) Aspect ratios from (D) and percentage aligned PLDCs from (E) plotted against the peak strain from (C) in each corresponding region of composites (represented by individual symbols).

Since PDLC alignment adjacent to dentin blocks appeared to be a biomechanical response to hydrogel swelling, finite-element analysis (FEA) was performed to simulate the mechanical behavior of swelling hydrogel composites. The FEA indicated that soft hydrogel swelling around a stiff, nonswelling dentin block created a radial gradient of tensile strains within the hydrogel, with the peak strains observed highest adjacent to the block and decreasing with increasing radius (Figure 3A,C). A strong linear correlation between the radially dependent tensile strains observed in the FEA and experimentally measured alignment (percent PDLCs with orientation angle 80–100° relative to dentin) (*R*^2^ = 0.95, *p* < 0.0001) or degree of PDLC elongation (aspect ratio) (*R*^2^ = 0.94, *p* < 0.0001) in discrete hydrogel regions suggested that a direct relationship between strain magnitude and PDLC orientation could exist above a certain strain threshold (Figure 3D–F). This modeled strain gradient is also in agreement with experimental studies of flexible substrates stretched around a rigid inclusion, which produces a similar strain gradient extending out from the inclusion.^23,24^ In contrast, the FEA simulation of a swollen hydrogel without a dentin block indicated a homogeneous distribution of low, radius-independent strain (Figure 3A). This finding, together with the random PDLC morphology present in hydrogels alone (Figure S1), further indicated that the PDLC alignment observed in the dentin–hydrogel composites was dependent on tensile strain.

**Figure 4:**
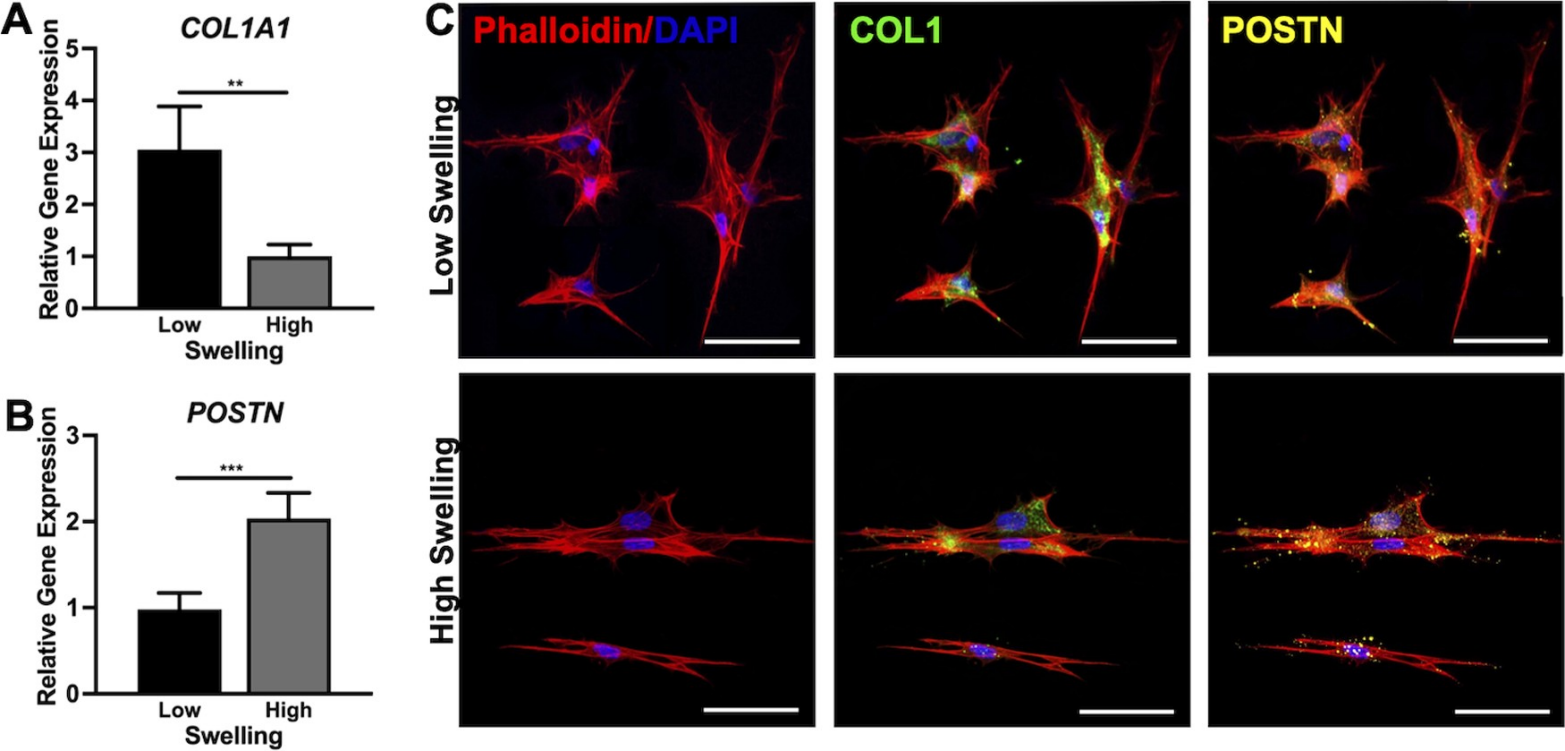
Relative gene expressions of *COL1A1* (A) and *POSTN* (B) at 7 days in randomly oriented PDLCs from low-swelling composites and aligned PDLCs from the central 4 mm diameter portion of high-swelling composites. Expression is normalized to the housekeeping gene *RPL32*. *N* = 3 hydrogels. Unpaired *t*-test. **p < 0.01, ***p < 0.001. (C) Representative confocal images of randomly oriented and aligned PDLCs stained for actin cytoskeleton (phalloidin, red), nuclei (DAPI, blue), Collagen type I (COL1, green), and Periostin (POSTN, yellow). Scale bars are 50 μm.

To investigate the influence of swelling-mediated strain on PDLCs, gene and protein expressions of two PDL markers, collagen type I and Periostin, were compared between regions of randomly oriented PDLCs within low-swelling composites (low strain) and aligned PDLCs in high-swelling composites (high strain). Collagen type I (*COL1A1*) gene expression was 3-fold higher in randomly oriented versus aligned PDLCs while Periostin (*POSTN*) expression was 2-fold greater in aligned PDLCs (Figure 4A,B). Both randomly oriented and aligned PDLCs showed intracellular collagen type 1 staining, while aligned PDLCs in high-swelling composites showed the presence of abundant extracellular Periostin (Figure 4C).

## Discussion

Cell alignment is a well-known response to strain. On two-dimensional substrates, cells react to cyclic uniaxial strain by aligning perpendicular to the direction of stretch, a behavior attributed to strain avoidance.^25,26^ How cells sense and respond to forces within three-dimensional materials is less understood. While cells can align parallel to the direction of stretch within collagen hydrogels, this process appears to be interdependent on cell-mediated collagen fiber alignment and occurs as collagen hydrogels contract rather than swell.^27−29^ Cells can also align within collagen hydrogels placed between dentin and bone blocks through similar mechanisms, but alignment is relegated to the central region, and cells show random or parallel orientation adjacent to block surfaces.^30^ Alternatively, cell alignment can be induced within synthetic hydrogel systems by incorporating materials such as magnetic cellulose nanoparticles^31^ or iron oxide nanoparticle-containing microgels^32^ but require the application of external magnetic fields to orient inclusions and provide contact guidance. Thus, the PDLC alignment within hydrogels demonstrated in this study appears to be a phenomenon distinct from previously reported models and occurs adjacent to the soft–hard tissue interface.

The PDL undergoes dynamic strain *in situ* during chewing and experiences static strain during orthodontic tooth movement, processes which concentrate stress at the PDL–tooth and PDL–bone interface and differentially activate resident PDLCs.^33,34^ Collagen type I is the primary component of the PDL fibers that transmits forces from tooth to bone. Compressive orthodontic forces, but not tension, stimulates collagen type I synthesis in PDLCs.^35^ Studies applying stretch to PDLCs in two-dimensional culture have also shown decreases^36^ or no change^37^ in the *COL1A1* expression. Periostin regulates collagen fibrillogenesis^38^ and is essential for maintaining the integrity of the PDL under mechanical loads.^39^ In contrast to *COL1A1*, tensile strain increases *in vitro* PDLC *POSTN* expression.^40,41^ Accordingly, the results of this study, where aligned PDLCs in high-swelling composites showed reduced *COL1A1* and elevated *POSTN* expression relative to PDLCs in low-swelling composites, further support the hypothesis that PDLC alignment in this system is a result of a swelling-mediated strain.

There are several avenues for further investigation based on the data reported herein. First, a more thorough understanding of how spatiotemporal swelling and strains develop within the hydrogel composites, as well as how cross-linked and free PEG arms contribute to hydrogel mechanical properties and nanostructure is necessary. Incorporation of techniques such as microparticle tracking-based strain mapping^42^ together with recently described theoretical models that account for hydrogel polymers with free arms^43,44^ may provide additional insight. The early impacts of swelling on encapsulated cells, where polymer densities, RGD concentrations, and strain levels are continuously shifting prior to reaching equilibrium, also require study. In addition, further work is required to investigate how mechanotransductive signaling pathways like calcium signaling and YAP/TAZ facilitate cell alignment in response to swelling-mediated strain.^45^

The current model also has certain limitations in replicating the periodontal complex, particularly the absence of a cementum layer to mediate the attachment of PDLCs to tooth roots. Within intact periodontal tissues, PDL fibers are inserted into both cementum on the tooth root and alveolar bone. During periodontal tissue formation, new cementum is first deposited on the root surface, followed by interdigitation of PDL collagen fibers with partially mineralized cementum collagen fibers that extend perpendicularly from the root surface,^46^ while PDL fibers insert into existing alveolar bone as it is remodeled.^47,48^ Engineering cementum formation on dentin surfaces followed by guided insertion and alignment of PDLCs likely requires multiphasic scaffolds and/or combinations of cells and signaling factors.^49^ Hydrogels with controlled swelling may act as a critical component of such an approach, utilizing strain to direct cell alignment relative to the tooth root and bone surfaces. For clinical applications, hydrogel swelling is beneficial in some situations such as soft tissue expansion^50^ but can be highly detrimental in others, compressing vital anatomic structures.^51^ Nevertheless, delivery of a construct containing a swelling or a preswollen hydrogel may represent a new approach for controlling the activity of transplanted cells.

## Conclusions

These results illustrate that PDLCs align perpendicular to dentin surfaces when cultured within swollen hydrogels, a novel finding enabled by the use of a synthetic polymer system through which hydrogel swelling could be controlled independently from other matrix properties. Swelling of soft hydrogels adjacent to stiff dentin substrates creates a gradient of tensile strain, which peaks at the dentin surface to induce PDLC elongation, alignment, and changes in gene and protein expression. Overall, hydrogel swelling-mediated strain holds potential as a tool for inducing cell alignment at soft–hard tissue interfaces.

## Supporting Information Available

PDLC behavior in swelling hydrogels without dentin blocks (Figure S1); PDLC viability and proliferation in dentin–hydrogel composites (Figure S2); PDLC alignment in the dentin–hydrogel composites with cylinders (Figure S3); PDLC alignment using different hydrogel molds and different block materials and/or cells (Figure S4) (PDF)
