# Supplementary material for: Hydrogel Swelling-Mediated Strain Induces Cell Alignment at Dentin Interfaces
Source: ACS Biomater Sci Eng. 2022 Jul 6;8(8):3568–75. doi: 10.1021/acsbiomaterials.2c00566 (PMC9364318; doi:10.1021/acsbiomaterials.2c00566)
Supplement: Supplementary file 1 — ab2c00566_si_001.pdf [file ab2c00566_si_001.pdf]

Hydrogel Swelling Induces Cell Alignment at Dentin Interfaces

David Fraser[1,2], Tram Nguyen[3], Alexander Kotelsky[3], Whasil Lee[3-5], Mark Buckley[3,5], Danielle S.W. Benoit[3,5-7]

1. University of Rochester, Eastman Institute for Oral Health, Department of Periodontology, Rochester, NY, USA
2. University of Rochester, Translational Biomedical Science, Rochester, NY, USA
3. University of Rochester, Department of Biomedical Engineering, Rochester, NY, USA
4. University of Rochester, Department of Pharmacology & Physiology, Rochester, NY, USA
5. University of Rochester Medical Center, Center for Musculoskeletal Research, Rochester, NY, USA
6. University of Rochester, Department of Chemical Engineering, Rochester, NY, USA
7. University of Rochester, Materials Science Program, Rochester, NY, USA

Corresponding author:
Danielle S.W. Benoit
Danielle.benoit@rochester.edu

Supplemental Materials

Number of Pages: 9

Number of Figures: 4

Number of Tables: 0

Supplemental Methods

1. Calculation of volume change and swelling ratios:

Fold-change hydrogel volume was calculated as the ratio of the swollen hydrogel volume (V_s) to the initial hydrogel volume (V_i) (equation 1):

$$\text{Fold-change volume } = \frac{V_s}{V_i} \tag{1}$$

Mass swelling ratio (q) was determined from the swollen hydrogel mass (M_s) and dry hydrogel mass (M_s)[1] (equation 2):

$$q = \frac{M_s}{M_D} \tag{2}$$

Volumetric swelling ratio (Q_V) was calculated as the inverse of the swollen polymer fraction $v_{2,s}$, where V_D is the volume of the dry polymer[2] (equations 3 and 4):

$$v_{2,s} = \frac{V_D}{V_S} \tag{3}$$

$$Q_V = \frac{1}{v_{2,s}} \tag{4}$$

2. Quantification of PDLC alignment in hydrogel-dentin composites:

ImageJ was used to select 4 sections (1000 μm x 2000 μm) of each whole hydrogel image corresponding to each side of the dentin block. Each section image was then rotated so the edge of the dentin block was at the top of the image, converted to 8-

bit, and then divided into 1000 μm^2 near (close to dentin) and far sections. The Directionality tool was then used separately for near and far sections with Fourier components method and nbins:45. The mean value for each bin in near or far region for the combined 4 sides was used as the value for each whole composite bin. These measurements were repeated for 3 composites per condition to give the mean ± standard deviation direction for each bin.

3. Finite element analysis

Finite element analysis (FEA) was implemented in FEBio[3] to simulate swelling of hydrogels with and without the embedded cylindrical dentin block and compare the resulting radial gradient of the tensile strains (i.e., radially measured 1st principal strains). Hydrogel swelling was induced by a 10 kPa effective fluid pressure prescribed on the boundary of hydrogel blocks. The hydrogel was modelled as a neo-Hookean biphasic material with Young's modulus of 3.5 kPa and Poisson ratio of 0.49[1]. A cylindrical dentin block (height: 2 mm, radius: 1 mm), embedded in the center of the hydrogel, was modeled as a neo-Hookean hyper-elastic solid with Young's modulus of 20 GPa and Poisson ratio of 0.3[4, 5]. Tensile strains observed in hydrogels with or without the inclusion of dentin were compared as a function of radial position from the center of the hydrogel or dentin cylinder. Note, that the effective fluid pressure implemented in FEA was not experimentally measured, however this quantity was consistently implemented in the two finite element models allowing for fair comparisons of the tensile strains. In addition, the FEA did not allow simulation of hydrogel swelling beyond a 2 mm radius without a distortion of finite elements leading to a lack of

convergence. Nevertheless, it is anticipated that the tensile strain gradient would be present at greater fold-changes in swelling volume and would also plateau to a level seen in hydrogels alone at increased radius.

Supplemental Figures

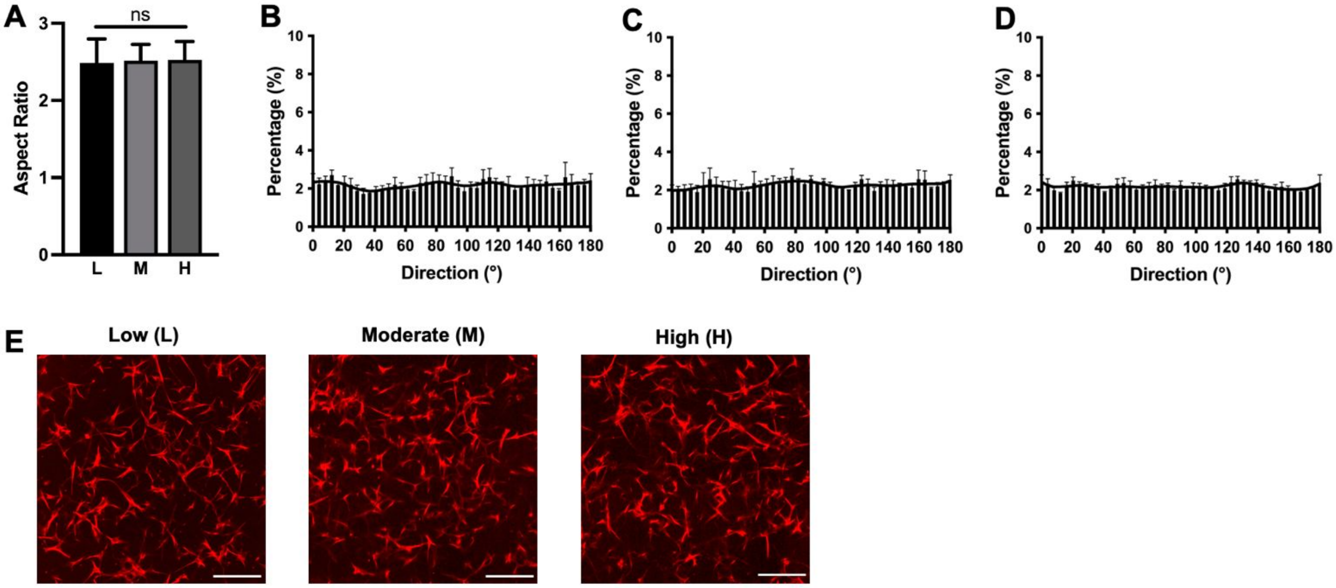

Figure S1. A) PDLC spreading (aspect ratio) in low (L), moderate (M), and high (H) swelling hydrogels. N=3 hydrogels. One-way ANOVA. B-D) Frequency distribution of PDLC alignment relative to an arbitrary plane set as the left border of the hydrogel image in B) low, C) moderate, and D) and high swelling hydrogels. N=3 hydrogels. E) Representative confocal images of PDLCs stained with Alexa Fluor 567 phalloidin (red). Scale bars are 200 μm.

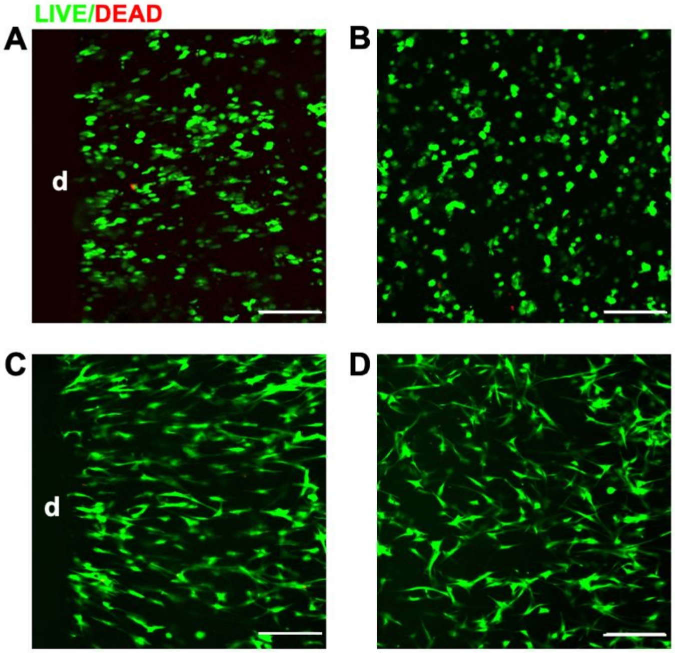

Figure S2. PDLCs in high swelling composites stained for live cells (Calcein AM – green) and dead cells (Ethidium homodimer-1 – red). A) Representative images of the region near the dentin block (d) and B) in the hydrogel periphery 1 day after hydrogel formation. C) Region near the dentin block and D) in the hydrogel periphery after 1 week of culture. Scale bars are 200 μm

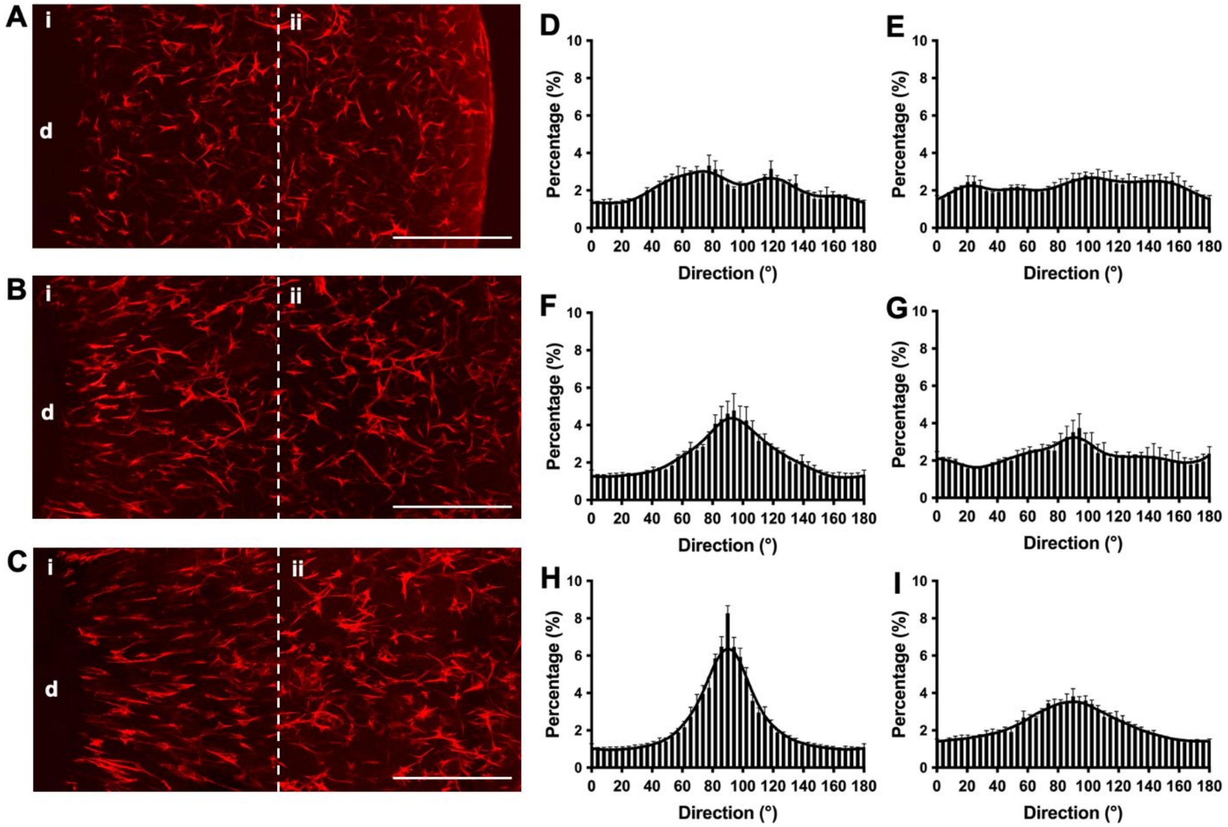

Figure S3. A-C) Low (A), moderate (B), and high (C) swelling composite with dentin cylinders (d) stained with Alex Fluor 568 phalloidin (red). Insets i and ii are representative images showing the near (i) and far (ii) regions where PDLC orientation was quantified. D-I) Histograms showing frequency distribution of PDLCs alignment relative to the dentin surface in near (D, F, H) and far (E, G, I) regions of low (D, E), moderate (F, G) and high (H, I) swelling composites. N=3 hydrogels per group. Scale bars are 1 mm.

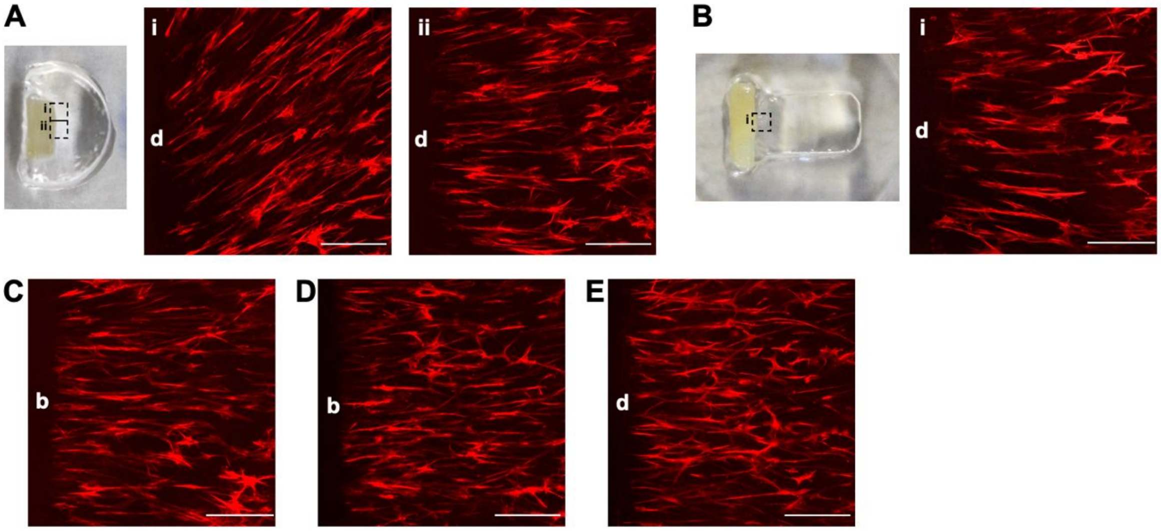

Figure S4. A) Photograph of swollen half cylinder composite with insets of stained PDLCs (i, ii) in the regions corresponding to dashed boxes. B) Swollen rectangular composite with inset of stained PDLCs (i). C) PDLCs adjacent to bone block (b), D) Bone marrow stromal cells (BMSCs) adjacent to bone (b), and E) dentin (d) blocks in high swelling composites. Scale bars are 200 μm.

References

1. Bryant, S. J. A., K.S., Photopolymerization of Hydrogel Scaffolds. In *Scaffolding in Tissue Engineering*, Ma, P. X. E., J., Ed. CRC Press: **2003**; pp 71-90.
2. Lutolf, M. P.; Lauer-Fields, J. L.; Schmoekel, H. G.; Metters, A. T.; Weber, F. E.; Fields, G. B.; Hubbell, J. A., Synthetic matrix metalloproteinase-sensitive hydrogels for the conduction of tissue regeneration: engineering cell-invasion characteristics. *Proc Natl Acad Sci U S A* **2003,** *100* (9), 5413-8.
3. Maas, S. A.; Ellis, B. J.; Ateshian, G. A.; Weiss, J. A., FEBio: finite elements for biomechanics. *J Biomech Eng* **2012,** *134* (1), 011005.
4. Qin, Q.-H.; Swain, M. V., A micro-mechanics model of dentin mechanical properties. *Biomaterials* **2004,** *25* (20), 5081-5090.
5. Kinney, J. H.; Marshall, S. J.; Marshall, G. W., The mechanical properties of human dentin: a critical review and re-evaluation of the dental literature. *Crit Rev Oral Biol Med* **2003,** *14* (1), 13-29.
